# What do editors do? Understanding the physiological functions of A-to-I RNA editing by adenosine deaminase acting on RNAs

**DOI:** 10.1098/rsob.200085

**Published:** 2020-07-01

**Authors:** Jacki E. Heraud-Farlow, Carl R. Walkley

**Affiliations:** 1 Cancer and RNA Laboratory, St Vincent's Institute of Medical Research, Fitzroy, VIC 3065, Australia; 2 Department of Medicine, St Vincent's Hospital, Melbourne Medical School, University of Melbourne, Fitzroy, VIC 3065, Australia; 3 Mary MacKillop Institute for Health Research, Australian Catholic University, Melbourne, VIC 3000, Australia

**Keywords:** ADAR, RNA editing, innate immune sensing, mouse models

## Abstract

Adenosine-to-inosine (A-to-I) editing is a post-transcriptional modification of RNA which changes its sequence, coding potential and secondary structure. Catalysed by the adenosine deaminase acting on RNA (ADAR) proteins, ADAR1 and ADAR2, A-to-I editing occurs at approximately 50 000–150 000 sites in mice and into the millions of sites in humans. The vast majority of A-to-I editing occurs in repetitive elements, accounting for the discrepancy in total numbers of sites between species. The species-conserved primary role of editing by ADAR1 in mammals is to suppress innate immune activation by unedited cell-derived endogenous RNA. In the absence of editing, inverted paired sequences, such as *Alu* elements, are thought to form stable double-stranded RNA (dsRNA) structures which trigger activation of dsRNA sensors, such as MDA5. A small subset of editing sites are within coding sequences and are evolutionarily conserved across metazoans. Editing by ADAR2 has been demonstrated to be physiologically important for recoding of neurotransmitter receptors in the brain. Furthermore, changes in RNA editing are associated with various pathological states, from the severe autoimmune disease Aicardi-Goutières syndrome, to various neurodevelopmental and psychiatric conditions and cancer. However, does detection of an editing site imply functional importance? Genetic studies in humans and genetically modified mouse models together with evolutionary genomics have begun to clarify the roles of A-to-I editing *in vivo*. Furthermore, recent developments suggest there may be the potential for distinct functions of editing during pathological conditions such as cancer.

## 1. Introduction

The epitranscriptome is the set of all biochemical modifications of RNA within the cell. In recent years, new techniques and reagents have allowed the genome-wide identification of modifications such as inosine, *N*^6^-methyladenosine (m^6^A), pseudouridylation, 5-methylcytosine (m^5^C) and more recently, *N*^4^-acetylcytidine (ac^4^C), among the more than 170 now described [1,2]. Following the cataloguing of sites, however, the task of assessing the biological importance and functions of the modified sites in both normal physiology and pathological states remains. Any one of the epitranscriptome modifications can number into the thousands or, in the case of inosine, millions of potentially modified sites per transcriptome. In this review, we will discuss the biological role of adenosine-to-inosine (A-to-I) editing within RNA in mammals and recent efforts to address the questions of how many sites there are, the editing enzyme responsible, which sites are essential and which functions are conserved across species?

## 2. Mammalian A-to-I RNA editing and adenosine deaminase acting on RNAs

Inosine is a highly abundant RNA modification produced by the deamination of adenosine within double-stranded regions of RNA (dsRNA). There are millions of A-to-I editing sites in the human transcriptome, with the majority in primate restricted to *Alu* elements [3–6]. In mice, there are 50 000–150 000 editing events, also concentrated in repetitive elements (SINE/LINEs) [7,8]. Because inosine is usually recognized as a guanosine by the ribosome, editing within protein coding sequences can change the amino acid codon and therefore the protein produced from the RNA [9,10]. An inosine base is decoded as guanine during RNA sequencing, resulting in A-to-G mismatches between the cDNA and genomic sequence, a feature exploited to allow genome-wide mapping [4,5].

As a result of the change to the RNA sequence, A-to-I editing can also influence splicing, RNA stability (through modification of miRNA-binding sites or other RNA-binding proteins), translation and localization [11–20]. Furthermore, editing has been reported to modify the biogenesis of non-coding RNAs such as microRNAs [21–23] and circular RNAs [24–26]. A-to-I editing within a transcript can occur at a single or isolated adenosine, termed site selective editing, or at many adenosines within an extended region, categorized as hyper-editing or editing enriched regions [27,28]. Initially discovered for its unwinding activity, the conversion of the adenosine base to inosine alters the base pairing properties within structured RNAs, changing the stability of the RNA secondary structure depending upon the context [29–31].

The proportion of RNA molecules edited at a given adenosine varies widely, from low and infrequent (less than 1%) to highly penetrant (approximately 100%). Editing rates can be different at a given site between tissues, developmental stage and cell type [32–34]. Across human tissues, arteries have the highest average editing level at coding sites, while editing at repetitive sites was broadly similar across the large number of adult human tissues in the GTEx collection [34]. The variability observed for editing at any given site can be partly attributed to both *cis* and *trans* regulation of A-to-I editing as well as abundance of the edited transcript and expression of the adenosine deaminases acting on RNAs (ADAR1 and ADAR2) [32,35]. Across mammalian transcriptomes the average editing frequency of all sites is less that 20% (i.e. less than 20% of the sequenced RNA/cDNA has a G in place of the genomically encoded A).

A-to-I editing sites are not evenly distributed across a transcript. In mammals, a small fraction of editing occurs in protein coding regions of transcripts, and there is evidence that this is evolutionarily conserved in a subset of sites and can alter the function of the resultant proteins [36,37]. This is best illustrated in a number of neurotransmitter receptors, where A-to-I editing is a key determinant of the functional potential of these proteins. The vast majority of editing sites, however, occur within intronic and untranslated non-coding regions containing repetitive elements, such as *Alu* elements in humans and SINEs/LINEs in rodents. This is thought to be due to the propensity of repeat elements to form double-stranded secondary structures that attract the editing enzymes, ADAR1 and ADAR2, that bind dsRNA through multiple dsRNA-binding domains.

Mammals express three ADAR proteins: ADAR1, ADAR2 and ADAR3 (encoded by *Adar*, *Adarb1* and *Adarb2*, respectively). ADAR1 and ADAR2 have both demonstrated editing activity *in vitro* and *in vivo* [38–40], while ADAR3 does not show editing activity *in vitro* and *Adarb2^−/−^* (ADAR3^−/−^) mice do not show alterations in editing [41,42].

### 2.1. Adenosine deaminase acting on RNA1 in health and disease

**Figure 1.**
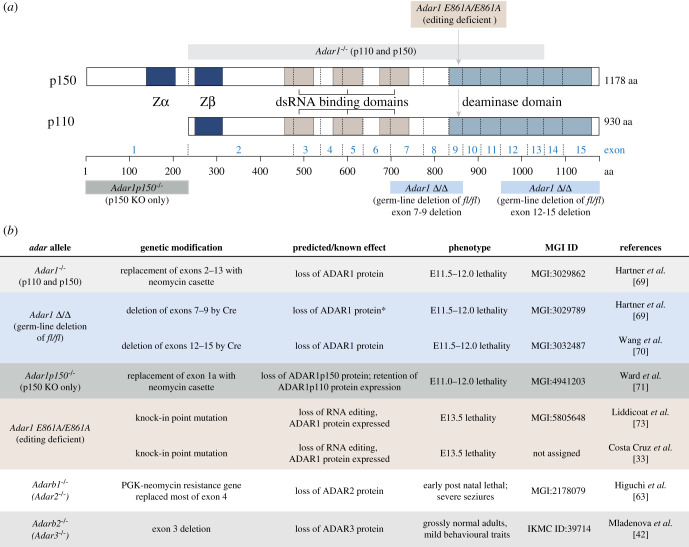
Summary of mouse alleles used to study functions of A-to-I editing. (*a*) Schematic representation of the two ADAR1 protein isoforms which are expressed from alternative promoters at the *Adar* locus and an indication of the various murine deletion alleles fall on the protein domain structure. (*b*) Summary of the different ADAR family mutant mouse models that have been described.

ADAR1 is widely expressed across cell types and tissues in both human and mouse. It is expressed as two isoforms, a constitutively expressed 110 kDa isoform (ADAR1p110) that is primarily in the cell nucleus and an inducible 150 kDa (ADAR1p150) protein that localizes to the cytoplasm. The ADAR1p150 isoform is lowly expressed basally compared with the p110 isoform but can be induced in response to a range of stimuli, most notably Type 1 interferon (IFNα/β) and pathogens that induce an interferon response such as infection with dsRNA virus [43]. While not covered in detail herein, a large body of work has defined roles for ADAR1 in the cellular response to viral infection including measles, HTLV and HIV-1, where it has both pro- and anti-viral actions (reviewed in [44]). While ADAR1p110 and p150 are both active editing enzymes, their key physiological functions may be distinct. This has largely been attributed to cellular location rather than unique functions in the nucleus compared with the cytoplasm, there is some evidence that ADAR1p150 may be more than a ‘cytoplasmic ADAR1p110’ [45,46]. The extended N-terminus of ADAR1p150 may contribute to these differences, including the additional Za domain (figure 1). It has been demonstrated that ADAR1p150 was a more efficient editor of known substrates than ADAR1p110 and the reasons and consequences of this are unknown [46]. Further work is required to understand the differences between ADAR1p110 and ADAR1p150 substrates and function.

Loss of function mutations in ADAR1 is principally associated with two diseases: dyschromatosis symmetrica hereditaria (DSH; OMIM phenotype 127 400) and Aicardi-Goutières syndrome (OMIM phenotype 615 010). DSH is not fatal and is characterized by small hyper- and hypo-pigmented skin macules. It is associated with heterozygous mutations in *ADAR* [47,48]. In contrast with DSH, Aicardi-Goutières syndrome (AGS) leads to profound disability and is often fatal [49,50]. Compound heterozygous *ADAR* mutations, predicted to be loss-of-function except G1007R which appears to be a dominant negative mutation, have been reported in patients with AGS [47,51]. AGS associated with *ADAR* mutation is designated AGS6 to differentiate these patients from alternative genetic causes of AGS, all of which appear to impact cytosolic nucleic acid sensing/metabolism or modification irrespective of the underlying gene mutation [50]. Intriguingly, given recent evidence suggesting differences between ADAR1p110 and ADAR1p150, one of the most common *ADAR* mutations in AGS6 is P193A, which would specifically impact the ADAR1p150 isoform. AGS is most often diagnosed in infants and is characterized by bilateral striatal necrosis in the brain with rapidly progressive developmental regression and dystonia. A feature of AGS is increased expression of interferon-stimulated genes (ISGs) in the peripheral blood and tissues, leading to the classification of AGS as an ‘interferonopathy’. AGS6 patients have significantly elevated expression of ISGs, a feature of ADAR loss of function that was first appreciated in the *Adar1-*deficient mouse models [52].

Recent studies have demonstrated that human cancers have increased A-to-I editing and ADAR1 levels [53–58]. This has been demonstrated across datasets and tumour types. There is no singular mechanistic basis proposed for the elevated ADAR1 expression; in some cases, there is genomic amplification of the *ADAR* locus while in other cancers there appears to be an active interferon response with leads to induction of *ADAR1* transcript expression. Several studies have linked increased editing of a specific substrate, such as AZIN1 in gastric cancers, to ADAR1 overexpression [53]. How generalizable a model where increased editing of a single substrate can account for the functional effect of elevated ADAR1 in cancer is not presently known. A plausible alternative hypothesis is that ADAR1 is elevated in cancers to facilitate immune evasion, in this case from the cytosolic innate immune system sensing of tumour genome-derived endogenous dsRNA. ADAR1 inhibition or loss has emerged as a potential therapeutic candidate for cancer and as a means to heighten the efficacy of immune checkpoint therapy [59–61] (discussed later).

### 2.2. Adenosine deaminase acting on RNA2 in health and disease

In contrast with ADAR1, ADAR2 is more restricted in its expression and is highest in the brain and central nervous system. It is expressed in peripheral tissues but is generally lower expressed than ADAR1 in these in both human and mouse [34,62]. Editing by ADAR2 has been historically associated with protein recoding, particularly of neurotransmitter receptors. The canonical example of ADAR2 editing is of the glutamine (Q) to arginine (R) recoding of the glutamate ionotropic receptor AMPA type subunit 2 (*GRIA2*). A-to-I editing at the Q/R site of the *GRIA2* transcript is near 100% efficient by ADAR2. The editing is required to form a functional receptor and the absence of editing at this single adenosine is fatal in mice due to the post-natal development of seizures [63,64]. This paradigm demonstrates the specificity and essentiality of A-to-I editing in the diversification of proteins from a fixed genome sequence. As our understanding and mapping of editing has evolved, there are multiple examples of ADAR2-dependent recoding events, some of which are evolutionarily conserved and under positive selection [36,37]. However, more recent data, discussed in more detail below, indicate that physiologically essential protein recoding is a rare event in mammals, where only GRIA2 editing is required under homeostatic conditions [37]. While ADAR2 expression correlates with overall recoding editing in large-scale human datasets from GTEx [34], there is accumulating evidence from mouse models that the ADAR1 editing of repetitive sites and ADAR2 editing of coding sites may be an inaccurate characterization [8,37].

Until very recently, no genetic variants/mutations in *ADARB1* (encoding ADAR2) had been associated with specific diseases. Bi-allelic *ADARB1* variants were found in patients with microcephaly, intellectual disability and seizures [65]. The mutations resulted in a range of changes in ADAR2 expression, with variants affecting splicing and isoform usage and others leading to reduced protein stability. The ADARB1 variants identified led to reduce editing activity of known substrates. Reductions in A-to-I editing have been reported in a range of diseases of the central nervous system, including autism spectrum disorders [66], seizures, epilepsy and psychiatric disorders [67]. It is not clearly ascertained that the reduced editing reported is specifically due to changes in activity of ADAR2, ADAR1 or both. Recent large-scale analysis of specific brain regions from schizophrenia patients and controls reported a more dynamic and nuanced picture, with reduced editing at some sites and increased editing at others [68]. There was evidence for genetic variance impacting editing, with approximately 30% of the editing events associated with a *cis*-regulatory element (termed editing quantitative trait loci or edQTLs). As brain region-specific and single-cell analyses are reported, the overall picture may become clearer.

## 3. The application of genetics to understand the *in vivo* function of A-to-I editing by adenosine deaminase acting on RNAs

Over the last 5–6 years, our understanding of the *in vivo* functions of A-to-I editing and ADARs in mammals has rapidly increased through the use of genetic models, predominantly murine models with modified human cell lines more recently being used. There are now multiple independently generated *in vivo* models of A-to-I editing deficiency that have clarified the functions of this epitranscriptomic modification and the enzymes that ‘write’ it in a living animal (figure 1). These models allow an understanding of the functions and contribution of each enzyme to mammalian physiology. Individual knockout models of each ADAR enzyme have been developed and characterized.

### 3.1. Murine *Adar* alleles

Five different *Adar* (encodes ADAR1) mutant alleles have been described to date (figure 1). Three models result in the loss of function mutations for both ADAR1p110 and ADAR1p150. One *Adar^−/−^* allele replaced exons 2–13 with a neomycin cassette, removing the bulk of the protein coding sequence [69]. Two *Adar*-deficient alleles have been generated through germ-line deletion of the loxP flanked exons, one resulting in the deletion of exons 7–9 (*Adar^Δ7-9^*) and the other removing exons 12–15 (*Adar^Δ12-15^*) [69,70]. Despite the difference in targeted region, the phenotypes of these germ-line mutants are highly comparable. The null animals die mid-gestation (E11.5–12.0), with evidence for failed foetal liver haematopoiesis.

The specific deletion of the longer p150 isoform of ADAR1 was achieved through deletion of the first exon (*Adar-p150^−/−^*) [71]. In this model, the expression of ADAR1p110 is retained. Strikingly, the *Adar-p150^−/−^* animals phenocopied the full deletion of ADAR1p110 and p150 with embryonic lethality at E12.0. This demonstrates that the essential physiological functions of ADAR1 are dependent on ADAR1p150 function, indicative of a key function for cytosolic editing or RNA binding.

Finally, specific inactivation of the catalytic deaminase domain through a point mutation (*Adar^E861A/E861A^*), where both the p110 and p150 proteins are expressed and can bind RNA but, not edit, have been independently generated in two laboratories [30,33]. While all mice with the deletion of *Adar* die around day E11.5-12, the editing-deficient point mutants were reported to survive slightly longer until E13.5. Both E861A mutant models phenotypically resemble the protein null alleles with foetal liver disintegration and a massive upregulation of interferon-stimulated genes. Given the point mutant and the *Adar-p150^−/−^* both show essentially the same phenotype as the full knockouts, it can be surmised that the A-to-I editing activity of the cytoplasmic p150 isoform is the essential physiological function of ADAR1.

**Figure 2.**
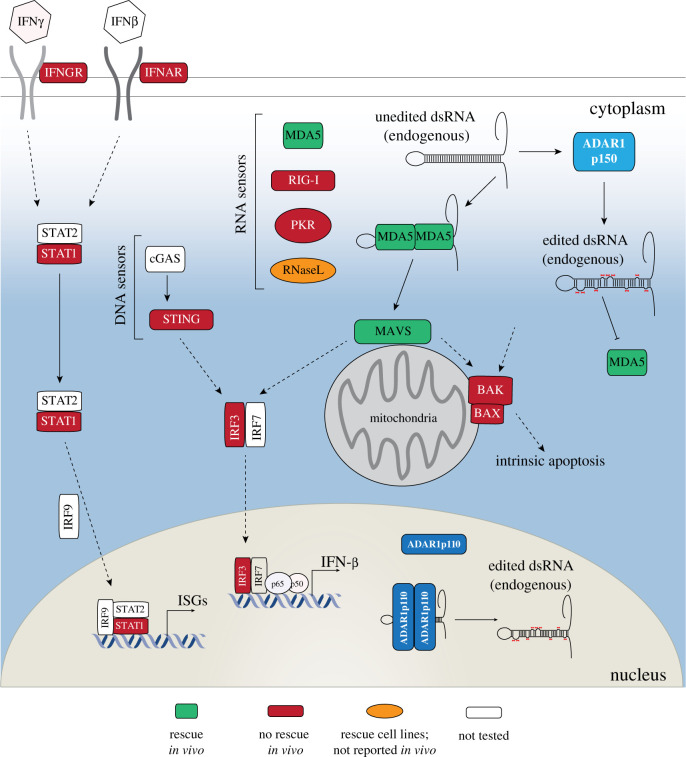
Summary of genetic crosses to test interactions with Adar. (*a*) Summary of innate immune pathways that respond to unedited dsRNA. Colour coded by whether co-deletion of the components rescue ADAR1-editing deficiency. See table 1 for detailed description of crosses and references.

In contrast with the elegant single adenosine replacement within the Q/R site of *Gria2* that rescued the *Adarb1^−/−^* (ADAR2-null) animals demonstrated 20 years ago [63], the *in vivo* biology of ADAR1 has only more recently been defined. There have been numerous reported crosses of the *Adar-*mutant alleles with other genes in attempts to understand cause of the embryonic death (summarized in figure 2 and table 1). These studies have focused on the profound upregulation of expression of ISGs in the ADAR1-null or editing-deficient animals and targeted both the interferon pathway components and the cellular cytosolic dsRNA sensing system. The conclusion from these *in vivo* analyses in the mouse, contributed to by multiple groups, is that deletion of the dsRNA sensor MDA5 (*Ifih1^−/−^*) or its downstream adaptor *Mavs* rescues all the ADAR1 mutants, albeit to varying extents (birth through to normal murine lifespan) [30,33,45,62,72,74]. This is the principal genetic pathway activated in response to a loss of ADAR1 expression or activity *in vivo*. The rescue was accompanied by the prevention of ISG activation, demonstrating that MDA5 is the physiological sensor of unedited self-dsRNA. The primacy of MDA5 as the sensor of endogenous dsRNA has been confirmed in human cell lines [45], and an alternative genetic cause of Aicardi-Goutières syndrome is gain of function mutations in *IFIH1* (encoding MDA5) [76]. Therefore, the species-conserved physiological function of ADAR1 is to edit endogenous dsRNA to prevent activation of MDA5.

**Table 1.** Summary of *in vivo* genetic analysis in mouse.

no rescue to birth *in vivo*					rescue to birth or longer *in vivo*				
*Adar* allele	test allele (protein name)	MGI allele (test)	viability	references	*Adar* allele	test allele (protein name)	MGI allele (test)	viability	references
*Adar1^−/−^* (p110 and p150)	*Stat1^−/−^* (STAT1)	not specified	E15.5	[72]	*Adar1^−/−^* (p110 and p150)	*Ifih1^−/−^* (MDA5)	3663677	die by day 2 after birth	[62]
	*Ifnar^−/−^* (IFNaR)	not specified	E14.5–E15.5	[72]		*Ifih1^−/−^* (MDA5)	not specified	lethal late embryonic, data not shown	[72]
	*Ifnar^−/−^Ifngr^−/−^* (IFNaR IFNgR)	1930950 (α) 1857286 (γ)	E15.5	[73]		Mavs^−/−^ (MAVS)	3799298	die by day 2 after birth	[72]
	*Trp53^−/−^* (P53)	1857263		J. Hartner & C.R.W. 2007–2008, unpublished data	Adar1^D/D^ (Ex7-9)	*Ifih1^−/−^* (MDA5)	3663677	most die by day 2 after birth	[45]
Adar1^D/D^ (Ex7-9)	*Tmem173^−/−^* (STING)	3817418		[45]		*Ifih1^−/−^* (MDA5)	3663677	median survival approximately 20 days	[74]
	*Ddx58^−/−^* (RIG-I)	3589395		[45]		Mavs^−/−^ (MAVS)	5313532	majority survival at least 20 days	[45]
	*Irf3^−/−^* (IRF3)	Not specified		[74]		Mavs^−/−^ (MAVS)	3654145	median survival approximately 15 days	[74]
Adar1^D/D^ (Ex12-15)	*Eif2ak2^−/−^* (PKR)	2182566		[70]	*Adar1p150^−/−^* (p150 KO only)	Mavs^−/−^ (MAVS)	5313532	majority survival at least 20 days	[45]
*Adar1^E861A/E861A^*	*Ifnar^−/−^Ifngr^−/−^* (IFNaR IFNgR)	1930950 (α) 1857286 (γ)	E15.5	B. Liddicoat & C.R.W. 2014–2015, unpublished data	*Adar1^E861A/E861A^*	*Ifih1^−/−^* (MDA5)	3663677	long-term adult survival	[30]
	*Bak1^−/−^Bax^−/−^* (BAK/ BAX)	2159364 (Bak) 1857429 (Bax)		[75]		*Ifih1^−/−^* (MDA5)	3801032	survival	[33]
					*Adarb1^−/−^* (*Adar2^−/−^*)	Gria2R/R (GluR-B)	2178125	long-term adult survival	[63]
					*Adar1^E861A/E861A^ Adarb1^−/−^* (ADAR1/2 editing deficient)	*Ifih1^−/−^ Gria2^R/R^*	3663677 (I) 2178125 (G)	long-term adult survival	[37]
						*Ifih1^−/−^ Gria2^R/R^*	3801032 (I) 2178125 (G)	long-term adult survival	[33]
					*Adar1^D/D^ Adarb1^−/−^* (ADAR1/2 editing deficient; Adar1DEx7-9 used)	Mavs^−/−^*Gria2^R/R^*	3654145 (M) 2178125 (G)	median survival approximately 18 days	[74]

The rescue of the ADAR1-null and editing-deficient animals has additionally highlighted differences between being protein deficient and having ADAR1 protein expressed, even if it can no longer edit RNA. The *Adar* knockout alleles resulting in a protein null state on an *Ifih1^−/−^* or *Mavs^−/−^* background survived at least to birth; however, none have reported to survive in significant numbers into adulthood (greater than 8 weeks of age) [45,72,74]. Importantly, this has been observed for independently derived *Adar* alleles, using different *Ifih1* and *Mavs* alleles and across multiple animal facilities. By contrast, *Adar^E861A/E861A^Ifih1^−/−^* animals are viable, fertile and survive to adulthood with no apparent defects in haematopoiesis or other tissues [30,62]. The ISG response, dependent on MDA5 and MAVS signalling, is near completely abrogated in the compound mutant mice consistent with this being an RNA editing-dependent phenotype [62]. The divergence in survival between rescued ADAR1-null and editing-deficient mice, however, may indicate editing-independent functions of ADAR1, especially in early post-natal development. Indeed, several groups have described potential ADAR1 editing-independent MDA5-independent functions of ADAR1 [45,77,78]. As yet, however, it is not clear why the knockout alleles have reduced survival compared with the editing-deficient mice when MDA5 or MAVS are co-deleted.

There are several additional findings from the *in vivo* genetic studies worth highlighting. First, the alternative cytosolic dsRNA sensor RIG-I cannot rescue the ADAR1 mutants, a finding confirmed orthogonally in human ADAR1-deifcient 293T cells [45]. This is important as it establishes that the unedited immunogenic endogenous dsRNA must conform to substrate preferences for MDA5. MDA5 has a preference for long(er) paired double-stranded RNA [79,80]. Second, the loss of ADAR1-mediated A-to-I editing does not cause cell death via the mitochondrial or intrinsic apoptosis pathway [75]. It is undisputed these cells die, but it is not via a BAK/BAX-dependent mechanism. Therefore, alternative forms of cell death cause lethality downstream of MDA5 and MAVS. Third, extracellular signalling via the Type 1 and Type 2 interferon receptors amplifies ISG activation in ADAR1-deficient embryos, but is not required for the activation of the innate immune response nor does neutralization of IFN receptor signalling prevent cell death as it can in other conceptually similar settings such as *Trex* deficiency [72,73,81]. These genetic studies have provided clarity to several of the key questions related to ADAR1 biology, particularly that MDA5 is the cytosolic sensor of unedited dsRNA. It remains to be determined which transcripts can become immunogenic when ADAR1 is not able to edit them and if these exact transcripts are conserved across species. The identification of endogenous RNAs that become immunogenic when unedited will prove important to understanding AGS and also in defining features of transcripts and secondary structures that can be potently immunogenic.

### 3.2. *Adarb1* alleles

*Adarb1^−/−^* (ADAR2^−/−^) mice die within three weeks of birth due to seizures. This lethality is rescued by the genomic substitution of a single editing site in the *Gria2* locus which is normally edited in 100% of the mRNAs produced from that locus [63]. The adenine to guanine point mutation mimics the edited inosine causing a Q to R substitution within the calcium channel of the glutamate receptor protein, GRIA2. Once this editing site was recoded genomically, the mice have normal neuronal calcium flux and don't develop seizures and the ADAR2 protein is dispensable for development. Extensive phenotypic analysis of the rescued *Adarb1^−/−^Gria2^R/R^* mice demonstrated subtle additional phenotypes including a change in hearing and immunoglobulin levels [82]. It has also been reported that there is a disruption in circadian rhythm in the *Adarb1^−/−^Gria2^R/R^* mice, although the consequences of this to the overall well-being of the animals is difficult to define [83].

### 3.3. *Adarb2* alleles

*Adarb2^−/−^* (ADAR3^−/−^) do not have any developmental defects [42]. They have recently been described to have increased anxiety and deficits in short- and long-term memory formation, which are dependent on hippocampus function. There was no significant difference in A-to-I editing in the brains from *Adarb2^−/−^* compared with controls, consistent with the lack of editing activity of ADAR3 *in vitro*.

### 3.4. No editing: Adenosine deaminase acting on RNA1/2 compound mutants

The single-mutant mouse models described above allow us to understand the contributions of each individual ADAR to normal physiology. These studies clearly delineated that the species-conserved function of ADAR1 is to edit endogenous dsRNA to prevent activation of the cytosolic innate immune response, with these unedited dsRNA being MDA5 substrates. ADAR2 is essential to recode the *Gria2* transcript to a functional glutamate receptor in the brain. However, there are 50 000–150 000 potential editing sites in the murine transcriptome and at least a magnitude more in humans. The potential for ADAR1 and ADAR2 to compensate for each other's functions in the respective single-mutant mouse remained a possibility. Indeed, transcriptome analysis of the individual single ADAR1 and ADAR2 mutants defined subsets of sites specific for each respective enzyme but the majority were edited at their normal level when either individual ADAR was absent [34,62]. This may be especially meaningful in the brain, where both enzymes are expressed, in contrast with many peripheral tissues where ADAR1 predominates. The establishment of the viable rescued ADAR1-editing-deficient mice enabled the genetic testing of this concept *in vivo*.

We recently reported the first completely editing-deficient animals where editing by both ADAR1 and ADAR2 were inactivated [37]. A second independent study generated *Adar^E861A/E861A^Ifih1^−/−^Adarb1^−/−^Gria2^R/R^* which were viable and aged normally [33,37]. Outside of an initially reduced body weight, which is attributable to the ADAR1^E861A/E861A^ as we had previous reported, no haematopoietic or histological phenotypes were found in these mice under standard housing conditions [37]. The surprising normality of completely A-to-I editing-deficient mice demonstrated that the majority of editing sites are not required for development to proceed normally. It is essential to emphasize that this is a conclusion drawn from the analysis completed to date of homeostatic animals housed under standardized conditions and does not exclude phenotypes becoming apparent with targeted testing or upon disruption of homeostasis. Moreover, the archetypal essential protein recoding site of GRIA2, which defined the role of A-to-I editing for decades, is the exception and not the rule. Bajad *et al*. [74] also generated an editing-deficient mouse where exons 7–9 of *Adar* were deleted *Adar^Δ7-9^Mavs^−/−^Adarb1^−/−^Gria2^R/R^* [74]. These had a similar median survival to the *Adar^Δ7-9^Mavs^−/−^* and *Adar^Δ7-9^Ifih1^−/−^* animals from the same group of approximately 18 days. The only difference being that the *Adar* mutants had reduced penetrance where a small number of long-term survivors were reported, whereas the additional loss of *Adarb1* made the lethality of the *Adar* allele fully penetrant with no long-term survivors reported [74].

Overall these data support the notion that the essential functions of ADAR1 and ADAR2 are non-overlapping in normal mouse development, a result that was somewhat surprising given the overlap of editing substrates between the two enzymes (detailed below).

## 4. How many editing sites are physiologically consequential?

Unlike many other modifications to RNA which require chemical modification or specific antibodies for detection, A-to-I editing is directly detected in sequencing data. This is because inosine is decoded as guanosine by the reverse transcriptase thereby producing A-to-G transitions in the resulting sequence. These can be detected as mismatches between the RNA sequence and matched DNA sequence or reference genome, or where available, mismatches between RNA sequence of wildtype animals and those with mutations in the editing enzymes *Adar* and *Adarb1*.

There have now been a number of studies seeking to catalogue the repertoire of editing sites both in mouse and human and across various tissues and developmental stages [6,8,34,37]. There are also several databases listing these sites, including RADAR and Rediportal [84,85]. These lists are heavily influenced by depth of sequencing, age of animal the sample was derived from, tissue used and filters (such as position of base in read, percentage editing level) to define an editing event. However, the use of the newly available Adar1/Adar2 fully editing-deficient mice now serve as an excellent control for false positives [33,37,74].

Analysis of the genetically controlled transcriptome datasets derived from whole brain of adult mice where the specific loss of ADAR1-mediated editing, loss of ADAR2 and loss of both could be compared resulted in several generalizable conclusions. First, there was no strongly enriched distinguishing sequence motif that could discriminate an ADAR1-specific site from an ADAR2 specific site in the mouse [37,74]. All sites essentially shared the same sequence context either side of the edited adenosine, consistent with previously defined sequence motifs [86]. Consistent with this, approximately 47% of the sites could be edited by either ADAR1 or ADAR2 [37]. Second, the analysis indicated that when considered transcriptome wide, there was a significant degree of overlap in the ability of ADAR1 and ADAR2 to compensate for each other in both recoding and repetitive editing regions [37]. When the subset of evolutionarily conserved editing events were assessed, which are enriched in protein recoding and neurotransmitter receptors, there is a greater dependence on ADAR2 specific editing that cannot be performed by ADAR1 [33,37]. Within this subet, there are also a small number of sites that are either competitively or coordinately regulated by the two ADAR proteins, which has been independently observed by others [33,37]. Licht *et al.* [8,10] recently used Nascent-seq which enriches for chromatin-associated RNAs and thus has higher coverage of introns. This method vastly increased the number of sites identified to over 90 000 novel editing sites in mouse brain. About 86% of these editing sites map to intronic regions and approximately 50 000 were lost in the *Adarb1^−/−^* brain suggesting ADAR2 may be a bigger contributor to intronic editing than ADAR1; however, *Adar*-mutant animals were not included in these analyses.

The use of genetic controls where one or both of the editing enzymes is disrupted has been useful for classing different types of editing sites [8,33,37]. Perhaps the most interesting outcome of this was the discovery that repetitive elements, which were previously thought to be selectively or at least preferentially edited by ADAR1 are equally well edited also be ADAR2 [8,37]. This raises the conundrum of why ADAR2 cannot compensate for ADAR1 to prevent activation of MDA5? There are several possible explanations for this. First, most hyper-edited regions are within introns, where repeats such as SINEs and LINEs can enable the formation of dsRNA structures preferred by ADARs, and only occur within the pre-mRNA. As these are spliced before nuclear export, editing by either ADAR1 or ADAR2 at these sites likely does not contribute to the potential immunogenicity of the RNA when cytosolic. Furthermore, many of the repetitive elements do not form ideal substrates for MDA5 (less than 300 bp and imperfect dsRNA structures) and are lowly edited, so the lack of editing is unlikely to directly activate an immune response [87]. There is evidence for strong purifying selection over evolution against long perfect dsRNA within mature mRNAs, providing further evidence that those within introns are less deleterious and may be more likely to be stochastically edited [87]. The second factor that probably contributes to the ADAR1-specific requirement to suppress immune activation is cellular compartmentalization. As most editing occurs co-transcriptionally, hyper-edited regions can probably be interchangeably edited in the nucleus by ADAR2 or ADAR1p110. However, a subset of dsRNA may, for example, be efficiently exported to the cytoplasm where ADAR1p150 is required to suppress innate immune activation. Finally, there is evidence that ADAR1p150 is more efficient at editing and there may be substrates that are more preferentially bound and edited by this isoform; however, this requires further investigation [46].

The extensive efforts to catalogue the number of editing sites across species now raises the question of which of these are important or phenotypically consequential. As discussed above, the mouse genetics supports the notion that many of these are not critical to organismal homeostasis. This is consistent with evolutionary genomic analyses of editing sites across species [36,87]. Barak *et al*. [87] studied the transcriptomes of 49 organisms and compared selection for inverted duplicated sequences (that can form long dsRNA) compared with tandem duplicated sequences (which do not form long dsRNA). There was evidence for very strong selection specifically against the inverted sequences in mRNAs. Interestingly, the selection against these sequences was far weaker in pre-mRNAs, arguing that those within introns don't pose a threat of self-activation of dsRNA sensors in the cytoplasm. Furthermore, the selection against both long and perfectly paired inverted sequences was so strong they are virtually absent. Coupled with the fact that these are also very lowly expressed, the findings suggest that strong purifying genomic selection is a first line of defence against self-dsRNA, where editing may be the back-up for newly arising sequences. Taken together, evolutionary genomics and mouse genetics both point to the common conclusion: that a large proportion of editing sites, particularly within repetitive elements, are incidental and of limited functional consequence.

Taken together, the complete absence of protein recoding outside of the single genomically engineered editing of GRIA2 (*Gria2^R/R^*) is well tolerated. It is a remarkable finding, given the number of conserved editing sites in receptors, that the removal of all editing of these is tolerated. Reciprocally, there does not appear to be any capacity for ADAR2 to reduce the formation of immunogenic unedited endogenously derived dsRNA which is an ADAR1-dependent function. The conclusion from this result is that while ADAR1 and ADAR2 can edit many common sites, they have non-redundant physiologically essential functions. A final conclusion from the *Adar^E861A/E861A^Ifih1^−/−^Adarb1^−/−^Gria2^R/R^* animals is that the majority of editing is dispensable for normal development and lifespan in the mouse.

### 4.1. Conservation of Adenosine deaminase acting on RNA1 function across mammals

The conservation of the biological response to loss of ADAR1 between human and mouse, despite the large difference in absolute numbers of potential editing events in each respective transcriptome (50 000–150 000 in mouse versus millions in human), is an important finding. Most editing in humans is in primate-restricted *Alu* repeat sequences, which comprise approximately 11% of the human genome and transcriptome, making it possible that editing in humans may be distinct to that in species lacking *Alu* elements. Mice do have an evolutionary ancestor of *Alu* elements (B1/B2 SINEs) and these are also subject to A-to-I editing [88]. The conservation of response across mammals could suggest that editing of species-conserved sequences is important or that the individual sequence is less relevant than the destabilization of a conserved secondary structure.

**Figure 3.**
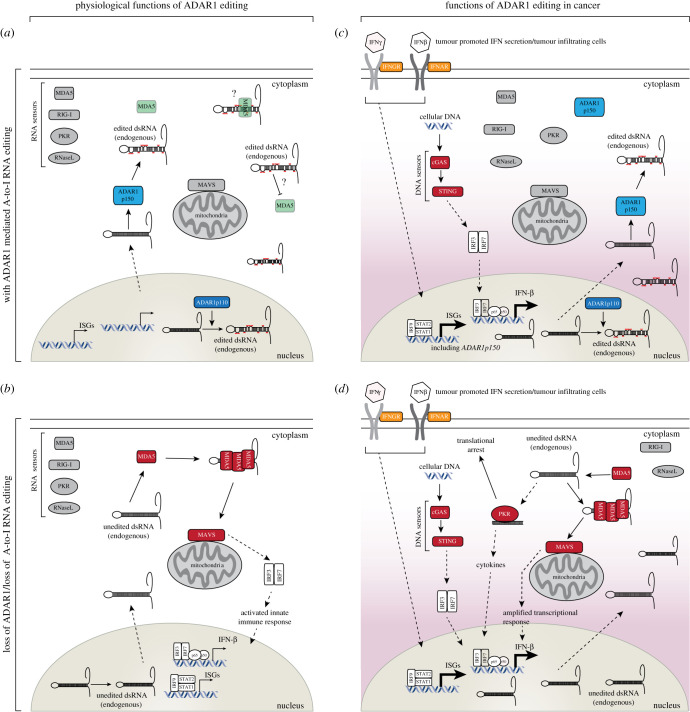
Model for alternative pathways responding to loss of editing by ADAR1 under physiological and pathological states. Under normal homeostatic conditions (*a*), low levels of dsRNA are produced by the cell, which are edited in both the nucleus and cytoplasm to prevent activation of MDA5. In the absence of editing (*b*), unedited endogenous dsRNA triggers the activation of MDA5/MAVS resulting in the production of IFN and ISGs. Some cancers have chronic activation of the DNA sensing innate immune pathway, cGAS/STING (*c*), which leads to increased interferon secretion, induction of ISGs including ADAR1 and PKR and increased dsRNA which is edited by ADAR1. In the absence of editing by ADAR1 in this state (*d*), the interferon-induced and cellular dsRNA triggers activation of both MDA5/MAVS and PKR leading to further induction of ISGs and translational shutdown. Abbreviations: IFN, interferon. ISGs, interferon-stimulated genes. In (*b,d*) the active pathway participants are coloured in red.

The culmination of mouse genetics centres on the role of editing by ADAR1 on the suppression of MDA5 activation by self-dsRNA. Co-deletion of other dsRNA sensors such as PKR or RIG-I did not modify the activation of interferon pathways in ADAR1 mutant mice [45,70]. By contrast, there is now considerable evidence that the loss of ADAR1 in human cells can lead to activation of PKR, and one report suggesting RNaseL [89], in addition to MDA5 [59–61,89–91]. This is particularly true in the case of interferon treatment, a preexisting active IFN pathway and cancer (figure 3). Similar to MDA5, PKR is a cytoplasmic dsRNA sensor that is activated in response to viral infection. Binding by dsRNA triggers PKR dimerization and autophosphorylation, leading to the phosphorylation of eIF2a and inhibition of protein synthesis [92]. It has previously been reported that ADAR1 can inhibit PKR activation in response to viral RNA [93]. Chung *et al*. found that ADAR1 KO in human cell lines lead to hyper-activation of PKR and translational shutdown in response to interferon treatment [90]. In this instance, both RNA-binding and catalytic activity of ADAR1 were required to inhibit PKR activation. Interestingly, the differentiation of human embryonic stem cells to neural progenitor cells led to the spontaneous production of interferon and activation of PKR. However, in this case, only knockdown of MDA5 could rescue the cells, whereas the knockdown of PKR had no effect, which is more consistent with the genetics defined in mouse (figure 2). From this, the authors proposed two distinct roles for ADAR1 in both the inhibition of MDA5 activation by self-dsRNA and the suppression of PKR-mediated translational shutdown in response to interferon. This is consistent with one study in mouse embryonic fibroblasts where PKR became activated in response to interferon treatment in *Adar^−/−^* and *Adar-p150^−/−^* cells but not *Adarb1^−/−^* or WT cells [91]. In parallel studies, Liu *et al*. identified a dependence on ADAR1 of a subset of primary tumours that chronically produce IFN, due to cell intrinsic activation of STING and DNA sensing immune pathways [61]. Due to the production of ISGs, which includes dsRNA sensors such as PKR, MDA5 and RIG-I, the tumours are particularly sensitive to the additional loss of ADAR1 which floods the cells with more dsRNA further activating immune pathways and cell death. A CRISPR suppressor screen found that PKR was required for the cell death induced by the loss of ADAR1 in the ISG-signature positive tumours. Similarly, an independent group found that a subset of lung cancer cell lines which have high ISGs are sensitive to the loss of ADAR1 [59]. Interestingly, the loss of MDA5 and MAVS suppressed the production of ISGs in the ADAR1-deficient cells but did not prevent cell death. By contrast, the loss of PKR partially rescued the lethality of ADAR1 deficiency. IFN treatment of ADAR1 KO-insensitive cells lines rendered these cells sensitive to loss of ADAR1 which was also at least partially dependent on PKR.

These distinct roles of MDA5 and PKR in ADAR1-deficient tumour cells was also reported by another group, where ADAR1 emerged as a candidate from an *in vivo* CRISPR screen to identify loss of function alleles that confer sensitivity of murine B16 melanoma cells to immune checkpoint blockade [60]. The loss of ADAR1 reshaped the tumour microenvironment leading to increased inflammation and growth arrest in response to IFN. This study also demonstrated that treatment with IFN increased the amount of editing and the ‘editing index’, suggesting that many of the transcripts induced as part of the response to IFN are themselves subjected to A-to-I editing. This leads to an increase in the cellular dsRNA load that may contribute to the involvement of PKR in these models. Consistent with the other studies, co-deletion of MDA5 was important to suppress the inflammation whereas the co-deletion of PKR prevented the growth arrest. However, it was only with co-deletion of PKR and MDA5 that the ADAR1-null tumours were no longer sensitive to immunotherapy. These studies have highlighted the interplay and potential cross-talk between nucleic acid sensors in the cytoplasm. Interestingly, in several cases, the expression of an editing-dead cytoplasmic ADAR protein is sufficient to rescue the phenotypes suggesting that a protein interaction between ADAR and PKR may play a role. Such cross-talk, particularly between ADAR1 and PKR, has previously been observed in the context of viral infection (reviewed by [43,44]).

Taken together, the current literature indicates that under certain circumstances PKR can be activated by a loss of ADAR1. Central to this genetic dependence appears to be prior activation of the cellular interferon response. The findings from various human tumour models point to distinct functions and/or interactions between MDA5 and PKR in ADAR1-deficient cells, where PKR is becoming activated in the context of an active interferon pathway. By contrast, it was tested with one of the initial Adar1 knockout mouse models that the loss of PKR did not rescue *Adar1^−/−^* mice [70]. Consistent with this, the rescue, to the extent that it has been tested, of the *Adar^E861A/E861A^* by concurrent deletion of MDA5 and suppression of ISGs in the rescued mice indicates that physiologically MDA5 is the sensor of unedited dsRNA [37,62]. These dichotomous results raise the possibility that the pathological requirement for ADAR1 and the genetic interactions observed may be distinct from those occurring physiologically. Based on these more recent findings, it would be interesting to test whether IFN treatment or viral infection of *Adar^E861A/E861A^Ifih1^−/−^* mutant mice would lead to translational shutdown and cell death via a PKR-dependent pathway. Whether this would be sufficient to provoke a response similar to that observed in ADAR1-deficient human cell lines treated with IFN would be able to be tested.

### 4.2. Drugging adenosine deaminase acting on RNA1?

One conclusion from studies in preclinical cancer models and the analysis of the tumour epitranscriptome is that therapeutic inhibition of ADAR1 is an attractive candidate for cancer therapy. From the available experimental data, this would be applicable to the specific subset of tumour types that have a cell intrinsic activation of the IFN response. An alternative application would be as a means to enhance response to immune checkpoint blockade. Enhancing immune checkpoint blockade response would have a potential broader action, enhancing cell intrinsic innate immune signalling in tumours and eliciting an immune response toward an otherwise immunologically silent/cold tumour. The perceived caveat of inhibiting ADAR1 is that the loss of ADAR1-mediated editing or of the protein isoforms completely is poorly tolerated *in vivo* based on the analysis of multiple murine loss-of-function models. It is worth reflecting on the case of the anti-apoptotic protein MCL1, where the somatic loss of function in mouse caused a rapid haematological failure that was fatal and led to the belief that inhibition may be too toxic for clinical development [94,95]. When small molecule MCL1 inhibitors were developed, it became apparent that the there was an achievable therapeutic window, highlighting key differences between small molecule inhibition and genetic ‘all-or-none’ models [96,97].

The response to an inhibitor of ADAR1, assuming that inhibition of A-to-I editing is the therapeutically desirable endpoint, would have expected side effects based on human genetic syndromes and the analysis of the murine models. Humans with compound heterozygous mutations in *ADAR* develop AGS6 and have a characteristic range of symptoms that ultimately prove fatal. It is relevant that not all human tissues are affected in AGS6 and many tissues and organs appear to function appropriately despite the reduction/loss of ADAR1 activity. This provides insight into the tissues that are either physiologically dependent or independent of ADAR1 *in vivo* in humans. In the mouse, somatic mutation models have demonstrated haematopoietic cells including primitive progenitors, B-cells and erythroid cells are particularly sensitive to the loss of ADAR1 as are other adult tissues such as the liver/hepatocytes (reviewed in [98]). Dependence on ADAR1 is presumably determined by the co-incident expression within individual cells of sufficient endogenous dsRNA ligand, MDA5 and ADAR1. If there is no dsRNA or MDA5 expressed then there seems to be no requirement for editing by ADAR1. This could be altered, however, by the addition of ligands that alter gene expression such as interferon, as seen in human 293T cells [90]. Widespread, non-cell-type-targeted somatic deletion of ADAR1 or somatic generation of ADAR1-editing-deficient adult mice resulted in a fully penetrant bone marrow failure and lethality [62]. These phenotypes could be rescued by the deletion of MDA5. This highlights that the potentially rate limiting consequences of an ADAR1 inhibitor on normal tissues would be mediated by MDA5, or what can be regarded as on-target toxicity of an ADAR1 inhibitor. The normal aging and histology of the rescued *Adar^E861A/E861A^Ifih1^−/−^* mice suggested that there are no essential protein recoding events mediated by ADAR1 *in vivo* that may be problematic. This view is further reinforced by the analysis of the completely A-to-I editing-deficient *Adar^E861A/E861A^Ifih1^−/−^Adarb1^−/−^Gria2^R/R^*, suggesting that on target toxicity would be limited to MDA5 activation with limited additional consequences, at least in normal tissues. The lack of apparent requirement for editing of the evolutionarily conserved editing events, particularly of neurotransmitters, may be advantageous to application of ADAR inhibitors in the adult context. However, toxicity profiles may differ between mouse and human given the substantial difference in abundance of repetitive elements between the species, where *Alu* elements are vastly more abundant in humans than SINEs/LINEs in mice. It is also worth considering if the effects of an adult mammal becoming editing deficient would be the same as what has been reported regarding the developmental and early post-natal phenotypes in the various mutant mouse models. Ultimately, this would require evaluation in tumours *in vivo* once inhibitors are available.

## 5. Concluding remarks

The last decade has seen a rapid advance in our understanding of the extent and breadth of A-to-I editing in mammals. Paralleling this has been the elucidation of key physiological functions of the ADARs and the identification of how ADAR1 links to the innate immune sensing system. The recent description of links between changes in ADAR function and disease––from neurological disease through to cancer––identifies new avenues for understanding how A-to-I modification of RNA can impact health and disease. The future holds the prospect of exciting new developments such as the development of small molecule inhibitors as well as utilization of ADARs to direct targeted *in vivo* RNA and gene editing [99], allowing full exploration of how A-to-I editing can be harnessed and manipulated to improve human health.

## Data accessibility

This article has no additional data.
